# Association of Juvenile Idiopathic Arthritis and Digeorge Syndrome; a Case Report 

**Published:** 2014-06

**Authors:** Farhad Salehzadeh, Amin Bagheri

**Affiliations:** Department of Pediatrics, Ardabil University of Medical Sciences, Ardabi, Iran

**Keywords:** Digeorge Syndrome; Juvenile Idiopathic Arthritis; Velocardiofacial Syndrome

Juvenile idiopathic arthritis (JIA) belongs to a group of arthritis with unknown etiology that occurs in children under 16 years old. Pathogenesis of this disease proposes the role of autoimmune process which is induced by antigens and results in inflammation of synovials and cartilage^[^^1^^]^. 

 DiGeorge syndrome (DGS) or velo-cardio-facial-syndrome (VCFS) is a genetic disorder due to a defect in 22q11.2 chromosome^[^^2^^]^. Patients with 22q11.2 DS usually have characteristic facies including retrognathia or micrognathia, long face, downturned mouth, short philtrum low-set, malformed ears and hypertelorism. Congenital heart defects, either a cleft palate or incompetence of the soft palate, and immune deficiencies are common. Patients may have short stature and occasional instances of growth hormone deficiency^[^^3^^]^. 

 Anomalies related to 22q11 monosomy have a wide range^[^^4^^]^. Renal, pulmonary, gastrointestinal, skeletal, and ophthalmologic abnormalities can also occur. Children and adults with 22q11.2DS have high rates of behavioral, psychiatric, and communication disorders. In children, these include attention-deficit/hyperactivity disorder, anxiety, and affective disorders. Adults have a high rate of psychotic disorders, particularly schizophrenia^[^^3^^]^. Parathyroid dysfunction may cause hypocalcemia and seizures in the neonatal period. Most patients with DGS have a partial form of the syndrome and thymic hypoplasia^[^^5^^]^.

 This defect results in cellular immuno-deficiency, although humoral defects have also been described. Autoimmune diseases have been associated with DGS, probably in consequence of T cell regulatory defects and impaired central tolerance^[^^5^^,^^6^^]^. These diverse immunoregulatory defects might predispose to the development of autoimmune disease such as hemolytic anemia, thyroiditis and inflammatory arthropathies^[^^5^^,^^6^^]^. 

 This study reports association of JIA and DGS in a 12 year old boy. He suffered from common cold like symptoms, overnight fever, weakness, and diffuse pain in limbs since 5 months ago. His right ankle had arthralgia and was swollen. Two weeks later his right wrist and left ankle also showed signs of arthritis. During the past two months he had intermittent fever with more joint involvement and disability which finally led to hospitalization of the patient.

 The patient is the first child of family. Because of cleft palate he had a surgery during infancy, and due to cardiac murmur, PDA was detected in echocardiography. He had normal developmental and intellectual process. His 10 year old sister had a palatoplasty when she was 2 years old because of cleft palate. In physical examination, he had T: 38°C, no skin rash and/or petechiae and purpura and ophthalmologic examination was normal. He had micrognathia clearly. A holosystolic murmur was heard over heart. A bilateral axiliary adenopathy in size of 2×1.5×2 cm was removed for pathologic study. 

 Positive Laboratory findings were: WBC: 15900 (Neut: 88%), Hgb: 10.6 g/dL, Plt: 549000, ESR: 70, CRP: 2+, Ca: 8 mg/dl, RF and ANA: negative and normal immunoglobulines. CT scan of thorax as well as sonography of abdomen and pelvis were normal. Radiography of the joints showed osteoporosis and soft tissue swelling. Lymph node biopsy revealed sinus histiocytosis pattern as a reactive adenopathy. Bone marrow aspiration was normal. Final diagnosis was oligoarthritic JIA. 

 With regard to cleft palate, micrognathia, and PDA, DGS was considered and genetic evaluation, Fish test, was done. Results showed elimination in 22q11.2, according to DGS. 

 Onset of JIA may be related to various types of chromosomal and genetic abnormalities with particular autoimmune process^[^^6^^]^. Association of VCFS syndrome with increased predisposition to JIA has been discussed by Rasmussen in three patients with polyarthritis and an evidence of impaired T cell function^[^^7^^]^. Two of the patients with polyarthritis also had IgA deficiency^[^^8^^]^. In a cohort study on patients with 22q11.2 deletion, etiologic relationship between DGS and JIA has been shown. JIA in this syndrome was 50 times more common than in normal population^[^^9^^]^. 

 Davies et al reported 5 new patients and analyzed 8 previously reported patients’ findings with the 22q11 deletion syndrome, who developed chronic inflammatory polyarticular arthritis. The arthritis in all these cases was moderate to severe, but at least partially responsive to methotrexate and/or corticosteroids, and was clinically indistinguishable from JIA. Of particular interest in his study was the high prevalence of IgA deficiency in this association^[^^10^^]^.

 Chromosome 22q11.2 deletion syndrome is associated with immunodeficiency, especially a mild to moderate deficiency in peripheral blood T cells. However, these patients could have normal laboratory evaluations^[^^11^^,^^12^^]^. Thymic hypoplasia or aplasia leading to defective T-cell function is the hallmark of DiGeorge anomaly. Depending on T-cell proliferative response to mitogens, DiGeorge anomaly can be classified as partial or complete. IL-7 may play a critical role in T-cell homeostasis in patients with partial DiGeorge anomaly^[^^13^^]^.

 Although DiGeorge anomaly is commonly associated with T-lymphocyte immunodeficiency, B-lymphocyte defects also occur. A recently published review of 1023 patients with DiGeorge anomaly revealed 6% of patients older than 3 years had hypogammaglobulinemia and 3% of patients with DiGeorge anomaly were receiving immunoglobulin replacement therapy^[^^14^^]^. 

 On the degree of T-cell deficiency patients have been divided into partial and complete forms of DGA. Whereas immunodeficiency was seen in the complete form, dysfunction of T-cell regulation was observed in milder forms of DGA, possibly related to abnormal T-suppressor cell function^[7]^. Peripheral blood may show the normal counts of blood T cell^[^^15^^, ^^16^^]^.

 The JIA disease is an antigen-driven autoimmune process, and despite HLA gene, inflammatory mediators are involved in this disease. It has been shown that the antigen-specific T-cells, especially T helper1 (Th1) are B cells, macrophages and monocytes stimulators, following that, pathogenic immune mediators induce disease process^[^^17^^]^. 

 Immunologic disorders in DGS especially T cell type abnormality with the main role of T cells on JIA immunopathogenesis, could explain association of these two diseases. 
